# Engineered bacteria: a novel era for metabolic, cardiovascular and cerebrovascular diseases therapy

**DOI:** 10.3389/fcvm.2026.1757348

**Published:** 2026-03-10

**Authors:** Yuzhe Wu, Ruiqi Sang, Xiangnan Chen, Songyun Wang

**Affiliations:** Renmin Hospital of Wuhan University, Wuhan, China

**Keywords:** cardiovascular disease, cerebrovascular disease, engineered bacteria, gut microbiota, metabolic diseases

## Abstract

There are a large number of people suffer from metabolic, cardiovascular, and cerebrovascular diseases, posing a significant threat to public health. Previous studies have suggested that cardiovascular and cerebrovascular diseases are frequently accompanied by changes in intestinal flora and metabolites, which play a crucial role in the progression of metabolic, cardiovascular and cerebrovascular diseases. Engineered bacteria, as a tool for efficiently expressing foreign genes within a bacterial cell line, has attracted considerable attention for cancer treatment over the past decades. In recent years, engineered bacteria has been extended to metabolic disorders, cardiovascular and cerebrovascular diseases therapy and has been a promising tool. However, several challenges persist, including the necessity of more refined strain selection and design. Furthermore, there is no relevant review on the application of engineering bacteria in the fields of metabolic diseases and cardiovascular diseases currently. Therefore, this review is aimed to explore the alterations of intestinal microbiota and metabolome associated with metabolic and cardio-cerebrovascular diseases, as well as the potential application of engineered bacteria in treating these conditions.

## 1 Introduction

**Figure 1:**
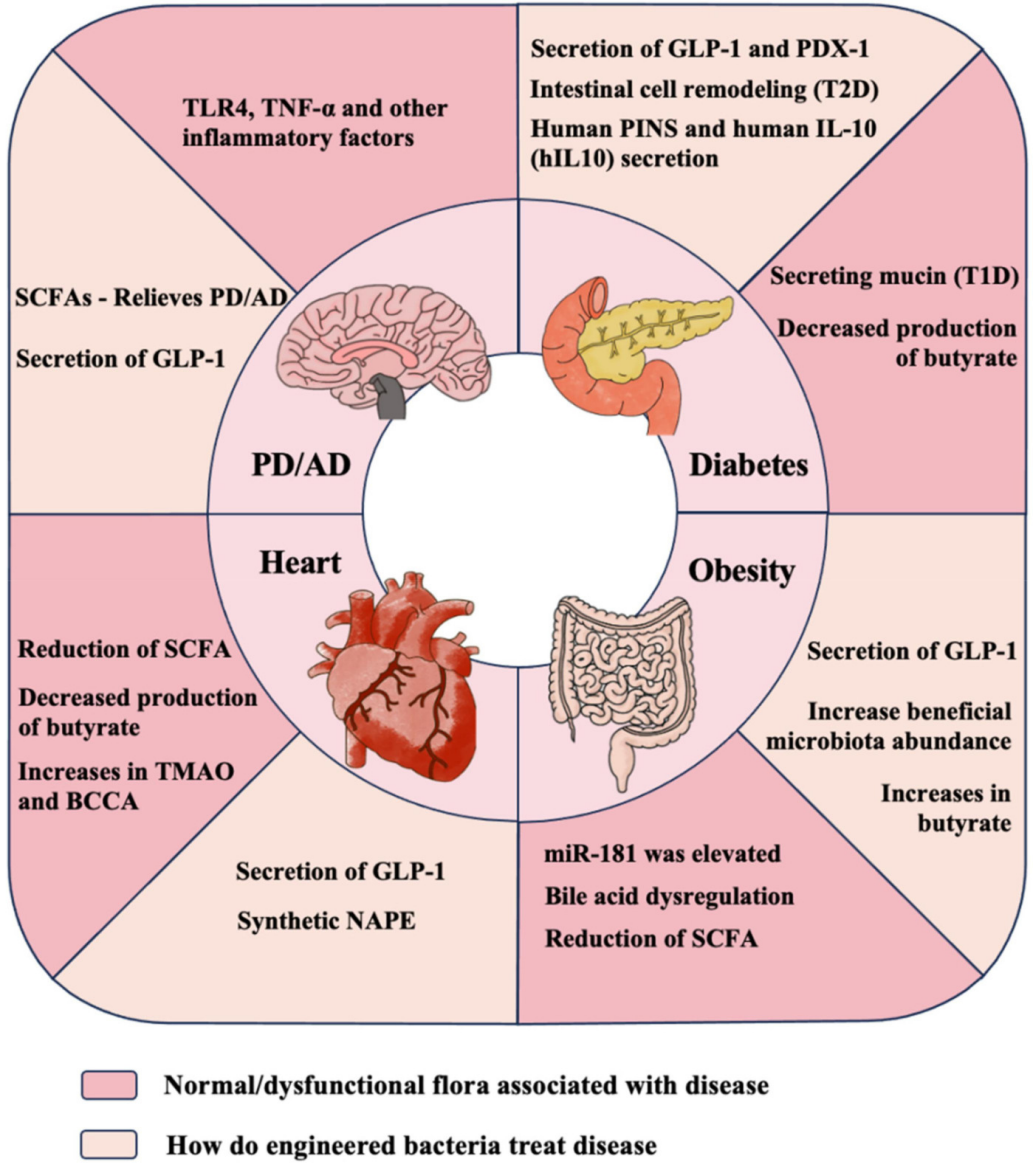
Rationale of engineered bacteria for metabolic and cardiac-carebrovascular diseases therapy. TLR, Toll-like receptors; TNF, tumor necrosis factor; GLP, glucagon-like peptide; SCFA, short chain fatty acid; PD, Parkinson's disease; AD, Alzheimer's disease; TMAO, Trimetlylamine oxide; BCCA, Branched chain amino; NAPE, N-acyl phosphatidylethanolamine; PINS, proinsulin; T1D, Type 1 diabetes; T2D, Type 2 diabetes.

As society progresses, there is a rapid growth in metabolic, cardiovascular and cerebrovascular diseases, which presents significant health and economic challenges. Earlier studies have demonstrated that gut microbiota and its metabolites plays a crucial role in the development of these diseases. Consequently, adjusting the microbiota's composition or its metabolites has become the focal point for therapeutic strategies. For instance, methods such as dietary adjustments, fecal microbiota transplantation, prebiotics, epigenetic modifications, and biosimilars can be used to maintain the diversity of intestinal microbes, diminish gastrointestinal inflammation, and safeguard the integrity of the intestinal barrier. Engineering bacteria has emerged as a significant and promising approach for disease modulation, particularly in fields like oncology, metabolic disorders, cardiovascular and cerebrovascular diseases. These bacteria, modified through advanced bioengineering techniques, can either be newly created or derived from natural or existing gut bacteria, transforming them into artificial microbes capable of enhancing the pre-existing biological flora. Such modifications enable these microbes to synthesize specific metabolites beneficial for treating pathological conditions by targeting the gut-brain and gut-heart axes. As shown in Figure 1. Additionally, these genetically tailored microbes can be finely controlled in a laboratory setting using modern techniques like near-infrared (NIR) light and ultrasound, allowing for precise adjustments to their activity and behavior.

Despite advances in clinical approaches, their limitations, such as prolonged treatment duration and the propensity for adverse effects and complications, have restricted their use in severe cases. Additionally, it has not been proven whether engineered bacteria might trigger an immune response. These factors highlight the need for improved interventions to enhance our treatment capabilities. Apart from these challenges, the use of engineered bacteria in these fields has not been well-reviewed. This paper provides a comprehensive overview of the current state of engineered bacteria in these conditions, identifying key methods such as genetic modification techniques and targeted delivery systems, and regulatory control mechanisms. Additionally, it discusses the unresolved issues including optimizing bacterial safety profiles and improving targeting precision.

## 2 Methods

For this narrative review, studies investigating the application of engineered bacteria in the treatment of metabolic, cardiovascular and cerebrovascular diseases were searched in the major databases of scientific literature (PubMed, ScienceDirect, Scopus) from those published between 1999 and 2025, using the keywords: gut microbiota, engineered bacteria, probiotics, cardiovascular diseases, coronary artery disease, hypertension, obesity, diabetes mellitus, Parkinson's disease, Alzheimer's disease, gut-heart axis, gut-brain axis, homocystinuria. Only relevant papers in English, performed with correct scientific design and published in peer-reviewed journals with good impact, were included. Case reports and congress abstracts were excluded, as were non-peer-reviewed papers. The manuscripts were reviewed independently by the three co-authors and the conclusions were shared and approved. Reference lists of selected articles were also analyzed, and occasionally cited papers were added to the review using the same criteria.

## 3 Engineered bacteria

Engineered bacteria, usually referring to bacterial cell lines that utilize genetic engineering methods to efficiently express exogenous genes, can be used to produce chemicals to improve the marine environment, produce biological products, and most importantly, as an emerging drug for diagnosing, preventing, or treating diseases. Engineered bacteria, probiotics, and prebiotics exhibit significant differences in regulating the gut microbiota to maintain cardiovascular health. Probiotics, as exogenous beneficial bacteria, exhibit unstable intestinal colonization. Prebiotics merely indirectly nourish endogenous beneficial bacteria without direct therapeutic effects. Engineered bacteria overcome these limitations through targeted genetic modification. Their controllable, targeted therapeutic effects outperform probiotics and prebiotics in cardiovascular disease intervention while also mitigating the issue of individual response heterogeneity in microbiota therapy (1).

As a special medical method, engineered bacteria can be used not only for diagnosing and detecting partial intestinal inflammation with their unique sensors (2), but also as drug delivery carriers for more precise anti-tumor treatments (3) and coronary artery disease (CAD) prevention (4). In tumor therapy, the mechanisms of action of engineered bacteria are diverse, but most of them are based on the basic condition of changing the genetic circuit of the bacteria or inducing their directional changes to target and colonize the confined growth in the vicinity of the tumor (5). Under this premise, engineered bacteria can be used to achieve therapeutic effects and improve remodeling of the local tumor microenvironment by intracellular delivery of therapeutic molecules (such as proteins) into (6) or out (7) of cancer cells, remodeling of the host immune environment through their own components (8), and in combination with other technologies such as MRI/PET imaging, Focused Ultrasound Surgery (FUS), magnetic guidance of nanoparticles, etc. (9). The therapeutic mechanism highlighted in this paper is an emerging one with greater relevance to metabolomics whereby engineered bacteria produce various types of metabolites through gene editing techniques to ameliorate metabolic disorders such as obesity and diabetes, to combat cardiovascular diseases such as hypertension and coronary artery disease, and to restore behavioral abnormalities and brain lesions associated with neurodegenerative diseases like Parkinson's disease (PD) and Alzheimer's disease (AD). These metabolites can be absent due to the patient's illness, neutralized by disease triggers, or used to improve existing local or systemic lesions. In addition, these bacteria themselves can act as general probiotics, regulating intestinal dysbiosis to reduce inflammation and improve the microenvironment. In the past few decades, based on the specific low oxygen and high enzyme environment of the intestine, most of the engineered strains are E. coli Nissle (EcN)1917 (10) and Lactobacillus (11). We have listed the applications of engineered bacteria in the treatment of various diseases in the table below Table 1.

**Table 1:** Therapeutic application of engineered bacteria.

Disease	Bacterial strain	Key mechanism	Main therapeutic strategy	Therapeutic effect	Ref
Diabetes mellitus	Lactobacillus gasseri ATCC 33323 (L)	Intestinal cells have been reprogrammed to produce insulin in response to glucose	Fasting for 6 hours, followed by STZ injection	Significant reduction in blood glucose	(12)
	Nissle	It secretes GLP-1 thereby activating insulin synthesis in pancreatic beta cells	Small intestinal crypt epithelial cells from IEC-6 rat treated with β cell protein	Stimulation of glucose-responsive insulin secretion	(13)
	GM L. lactis	Targeting PINS and IL-10 to the intestinal mucosa,then combined with low doses of Anti-human CD3 mAb	Hamster anti–mouse CD3 mAb	Autoimmune destruction was reduced	(14)
Obesity	Nissle 1917(EcN) and At1g78690 (pNAPE-EcN)	Hepatic NAE level and the fatty acid oxidation genes in hepatic mRNA expression were increased	A daily dose of 11CFU pNAPE-ECN or pEc bacteria was given to lean male C57BL/6J mice	Inhibition of weight gain and obesity;significantly improved glucose tolerance; Reduced insulin resistance index and increased insulin response observed in high-fat diet feeding mice	(15)
	Lactococcus lactis	PPARα and its target genes are up-regulated; Increased expression of fatty acid oxidation genes; Reduced expression of proinflammatory cytokines	C6BL/5 male mice was fed with PBS,MRS medium containing 109 M-GLP-1	Significantly reduced the body weight, adipose tissue weight and liver lipid accumulation; Improved glucose intolerance, liver function and the expression of fatty acid oxidation related genes	(16)
	Nissle 1917(EcN-GM)	Modulating neuropeptide level related to hypothalamic homeostatic control	Specific no-pathogen mice was gavaged with 0.3 ml of 10^10^ CFU/ml control EcN, EcN-GM (genetically engineered EcN) and 10 ml/kg orlistat.	Weight and food intake were reduced; Reduced the blood glucose levels, total cholesterol and liver triglyceride content; Improved hepatic steatosis and fibrosis	(17)
	B. subtilis SCK6	Enhanced butyric acid production to supplement BA	Male C57 BL/6J mice was fed with engineered *Bacillus subtilis* SCK6	Improved insulin response and regulates blood glucose; Reduced hepatic steatosis and fat accumulation	(18)
PD	E. coli Nissle 1917	GLP-1 activate the PI3K/protein kinase B (AKT) pathway and inhibit the downstream of the NF-κB signaling pathway to inhibit the expression of pro-inflammatory cytokines	Male C57BL/6 mice injected MPTP intraperitoneally was fed with EcN-GLP-1	Improved motor coordination and reversed pathological changes; Inhibited brain inflammation, the homeostasis of intestinal flora and colonic inflammation in PD mice;	(19)
	Lactococcus lactis subsp. cremoris	Significantly reduced the expression of TLR-4, p-NF-κB in the NF-κB signaling pathway, and the expression of IL-1β, IL-6 and TNF-α at gene and protein levels	Male C57BL/6 mice injected MPTP intraperitoneally was fed with MG1363-pMG36e-GLP-1 strains in drinking water	Improves exploratory and motor functions in mice with Parkinson's disease; significantly inhibited the activation of astrocytes and microglia;	(20)
	C. butyricum (NCU-02, CGMCC, no. 25504	GLP-1 is secreted to activate the mitophagy pathway; Increased the activity of SOD and GSH-Px; Inhibited the activity of MDA against OS	C6BL/22 injected MPTP intraperitoneally received C.butyricum-GLP-1 gavage	Improve motor dysfunction; Alleviate nerve pathological changes; Improve intestinal flora homeostasis;	(21)
Cardiometabolic Disease	EcN (Ardey Pharm, GmbH)	Increased expression of Aco and Cpt1a; Reduced hepatic expression of Cd36, Acc1 and Acc2; Activate PPARα and GPR119 to mediate the anti-atherosclerotic effects	C57BL/6J wildtype mice was fed with E. coli, Nissle 1917 (EcN) in drinking water	Inhibition of weight gain; Increases hepatic and adipose NAEs; Inhibits the accumulation of liver TG and reduces hepatic inflammation and fibrosis; Reduces atherosclerotic lesion necrosis	(22)
High blood pressure	Clostridium butyricum	Activate ACE109, AMPK/mTOR/p70S6 K/4EBP1 signaling pathway; Secreted GLP-1 binding with GLP-2R in the kidney and regulates the RAAS system;	Spontaneous hypertensive rats (SHRs) received intragastric administration of CB-GLP-1.	Improved High blood pressure; Improved ventricular hypertrophy; Improved the homeostasis of intestinal flora	(23)

STZ, streptozotocin; GLP, glucagon-like peptide; IEC, intestinal epithelial cell; PPARα, peroxisome proliferator-activated receptors; AD, Alzheimer's disease; AgRP, agouti-related protein; NAPE, N-acyl phosphatidylethanolamine; GM, genetically modified; NPY, neuropeptide Y; POMC, proopiomelanocortin; HDAC, histone deacetylase; NF-κB, nuclear factor kappa-B; α-Syn, α-Synuclein; MPTP, 1-methyl-4-phenyl-1,2,3,6-tetrahydropyridine; GFAP, glial fibrillary acidic protein; TLR4, toll-like receptor 4; IL-1β, interleukin-1β; IL-6, interleukin-6; TNF-α, tumor necrosis factor; SOD, superoxide dismutase; GSH-Px: glutathione peroxidase; MDA, malondialdehyde; OS, oxidative stress; PINK-1, parkin mediated mitophagy-1; GPR, G protein-coupled receptors; TG, triglyceride; AMPK, adenosine 5’-monophosphate-activated protein kinase; mTOR, mammalian target of rapamycin; NAEs, N-acyl ethanolamides; RAAS, renin aniotension aldosterone system.

## 4 Role of intestinal flora and its metabolites in metabolic diseases and regulatory strategies for engineered bacteria

Maternal gut microbes rapidly colonize the infant during labour and become the gut microbes of the offspring (24). Postnatally, microbial composition is regulated by factors such as host diet, hygiene and antibiotics (24). In humans, this change usually stops slowly about 5 years after birth, reaching a relatively stable state in the absence of strong external disturbances (25). Previous studies have shown that gut microbes have a large impact on host health, including BMI, blood pressure, HbA1c and depression (26). In metabolic disorders, especially in cases of Nonalcoholic Fatty Liver Disease, researchers have documented substantial changes in the composition of gut microbiota. Specifically, there has been a noticeable reduction in the populations of certain phyla known for their thick cell walls, coupled with an increase in the prevalence of bacteria from the Anaplasma genus (27). These findings illuminate the intricate ways in which our gut's microbial inhabitants are intertwined with the host's metabolic pathways, shedding light on the potential role of these microorganisms may play in influencing the onset and progression of metabolic diseases. In addition, the metabolome of microorganisms often serves as an important factor indirectly influencing metabolic and biochemical activities in the host, but it is important to note that as clinical interventions such as antibiotic use and near-infrared (NIR) therapy become more prevalent, they may affect microorganisms and the disease phenotype (28).

### 4.1 Obesity disease

Obesity can lead to a variety of comorbidities including coronary heart disease, fatty liver disease, diabetes, etc., which seriously affects human health and quality of life. An experiment conducted in 2005 has showed that mice with the obesity gene had significant changes in their gut microbes (29). Over the past number of years, a substantial number of studies have demonstrated that obesity is strongly associated with the gut microbes. On one hand, in obese populations, body weight is negatively correlated with the diversity of gut microbes, and the abundance of Mycobacterium spp. increases after weight loss (30). On the other hand, gut microbes can regulate the expression of the microRNA family miR-181 in white adipose tissue, which promotes host obesity by reducing the production of the metabolite indole and increasing miR-181 (31). Intestinal microbes regulate host body weight by producing metabolites such as short-chain fatty acids (SCFAs) and bile acids (32).

**Figure 2:**
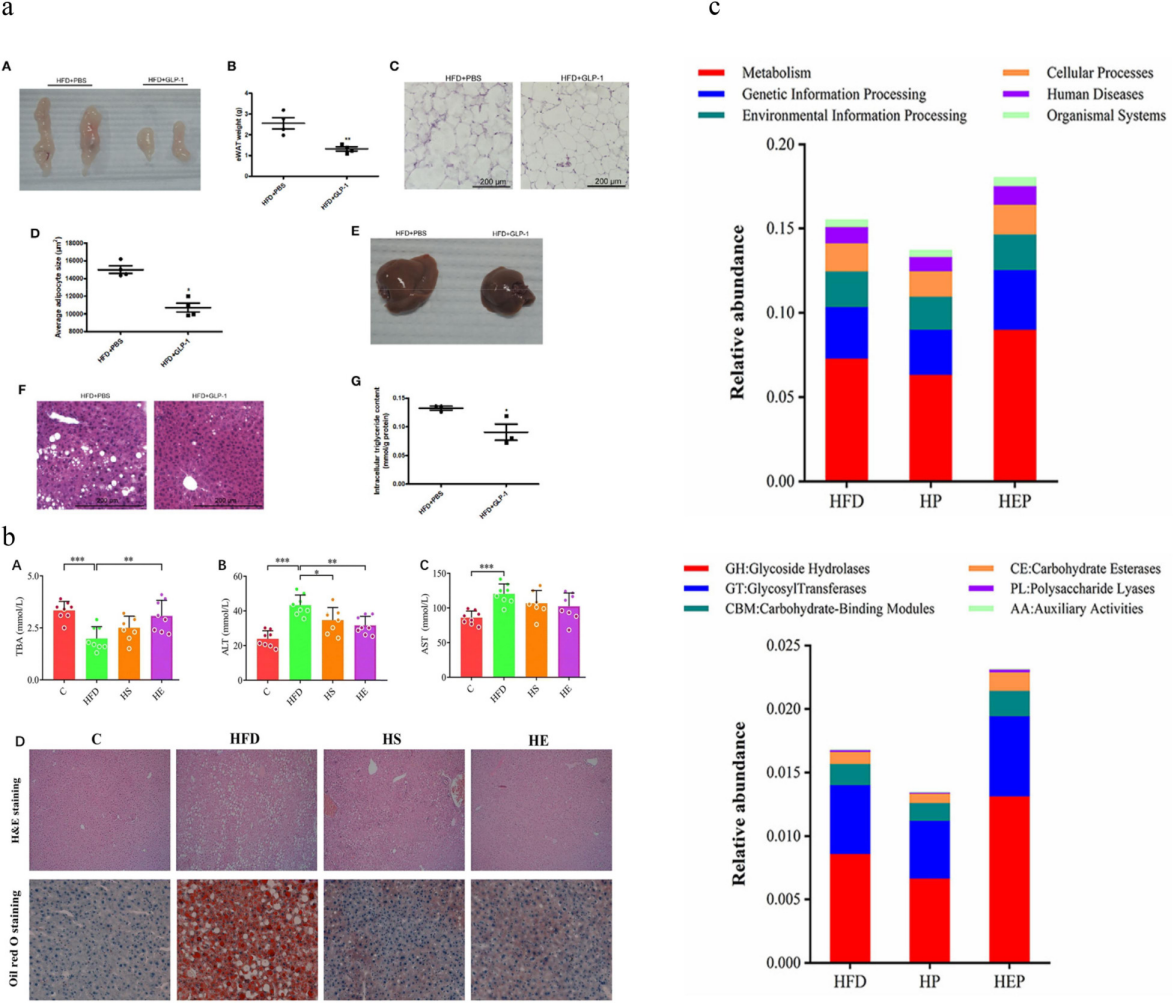
**(a)** The epididymal tissue weight of HFD mice treated with M-GLP-1 showed improvements in adipocyte size, number of steatosis hepatocytes, liver lipid deposition, and TG levels. Reproduced with permission from Ref. (34). Open Access. **(b)** BsS-RS06550 reduced fat content and ameliorated hepatic steatosis in HFD-fed mice. From Ref. (17). Open Access. **(c)** Functional relative abundance of intestinal flora based on the KEGG database. The engineered bacteria (B. subtilis BsS-RS06551) significantly influenced the growth of dominant intestinal bacteria in mice fed high-fat diets (36). Open Access.

In a cross-sectional study of Japanese adults, the symbiotic bacterium B.exlerae improves obesity by reducing the number of macrophages and promoting the consumption of adipose tissue (33). Obesity is associated with white adipocytes, and glucagon-like peptide-1(GLP-1) can promote adipocyte differentiation by regulating signaling pathways. In humans, GLP-1 not only stimulates insulin secretion in a glucose-dependent manner, but also suppresses glucagon, delays gastric emptying, reduces appetite and induces satiety, leading to weight loss (34). Release of GLP-1 from M-GLP-1 engineered bacteria in mice showed improvements in adipocyte size, number of steatotic hepatocytes, liver lipid deposition, and TG levels (35) (Figure 2a). The production of engineered bacteria compensates for the lack of the potent anorexic lipid family N-acyl phosphatidylethanolamine (NAPEs) (15). Additionally, butyrate produced by engineered bacteria effectively treats obesity by promoting energetic expenditure (36). Reduced the level of butyrate in the intestinal tract may result in the exacerbation of local inflammation, ecological imbalance, and impairment of intestinal barrier function (37). The mechanism by which butyrate acts on obesity is associated with its ability to activate AMPK, increase ATP consumption, and induce PGC-1α activity. Butyrate stimulates mitochondrial function by upregulating the expression of genes involved in lipolysis and fatty acid β-oxidation, which constitutes its molecular mechanism of action (36). Liang Bai's team genetically modified Bacillus subtilis SCK6 to improve the yield of butyrate, and detected a decrease in fat content and liver steatosis in mice, regulating serum chemical indices (18) (Figure 2b). Lina Wang further validated that engineered bacteria can effectively influence the growth of dominant intestinal bacteria, such as bifidobacterium and Lactobacillus, changed the structure of the flora and affected the metabolites in the host (38)(Figure 2c).

### 4.2 Diabetes

Type 1 diabetes (T1D) is primarily caused by the destruction of pancreatic beta cells due to genetic, immunological and other factors. In contrast, Type 2 diabetes (T2D) typically arises from inadequate insulin secretion or decreased insulin sensitivity in target tissue cells, resulting in impaired glucose and lipid metabolism in the body. Increased small intestinal permeability in T1D has been demonstrated (39). Some bacteria increase mucin degradation, results in reduced integrity and increased permeability of the intestinal mucosa, allowing bacterial translocation and stimulating the immune system to produce antibodies that cross-link with pancreatic islet β-cell surface antigens, thereby destroying the cells and leading to T1D (40). Butyrate, a major end-product of gut microbes, maintains the intestinal mucosal defense barrier by selectively up-regulating tight junction proteins and regulating energy homeostasis (41). Considerable research has shown that diabetic patients have a disproportionate ratio of flora in their bodies, with lower numbers of Bifidobacterium, Escherichia coli and Lactobacillus in diabetic patients, and a significant increase in the relative abundance of Clostridium sporogenes (42, 43).

**Figure 3:**
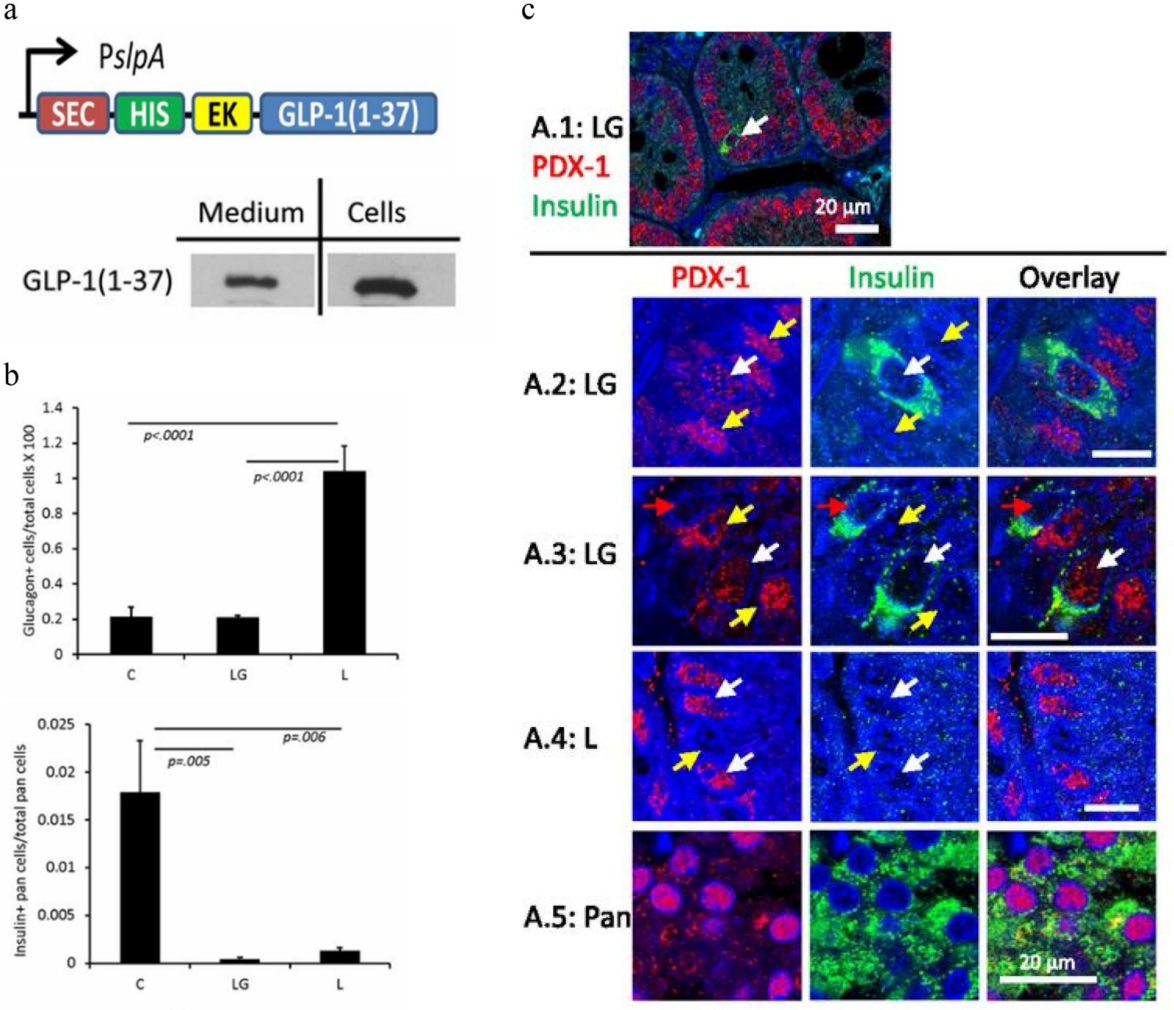
**(a)** Lactobacillus gasseri ATCC 33323 **(L)** was designed to secrete GLP-1 (1–37), and was confirmed in culture. **(b)** The ratio of glucagon-positive cells to total pancreatic cells was higher in L-treated rats, and the rats in this group had more remaining beta cells. **(c)** PDX-1 production was observed in the upper intestine and pancreas.Reproduced with permission from Ref. (11). Copyright 2015, American Diabetes ASSociation.

Due to the differences in the pathogenesis of T1D and T2D, addressing the diversity of their etiologies can provide different therapeutic strategies. Following an *in vitro* experiment of EcN 1917 was engineered to secrete GLP-1 or full-length pancreatic and duodenal homeobox gene 1(PDX-1) protein after gene editing by Faping Duan (13), his colleagues followed up with *in vivo* experiments using Lactobacillus garrii ATCC 33323 (L) as the parent. Engineered bacteria that can secrete GLP-1 (1–37) were generated and tested *in vivo* in rats (Figure 3A). In the experiment, the intestinal epithelial cells of the rats underwent remodeling to secrete insulin, as well as β cell markers such as PDX-1, MafA, and FoxA2, thereby reducing blood glucose levels of the rats (Figures 3b,c) (12). Tatiana Takiishi's team, in contrast, proposed a different approach by modifying the bacterium so that it can secrete cytokines such as Interleukin-10 (IL10), which can lead to an immune response in the gut (14). Specifically, the Lactobacillus lactis strain was genetically engineered to produce human proinsulin (PINS) and human IL-10 (hIL10), named GM L. Lactis. The mechanism of action is related to the immune response; specifically, the gene-edited engineered Lactobacillus lactis can target PINS and the biologically active immunomodulatory cytokine IL-10 to the intestinal mucosa and bind to low doses of anti-CD3 monoclonal antibodies, resetting the immune system to develop β-cell-specific tolerance. Similarly, Lacticoccus lactis was produced by Sofie Robert et al. through the transformation of MG1363 strain, or IL10-secreting sAGX0037 strain with the GAD65370-575 encoding plasmid, which was combined with a short course of low-dose anti-CD3. It can even reverse the course of disease in NOD mice with recently developed diabetes (44). Li Cuiying's EcN-pGEX-GLP-1 GM strain also indirectly reduced diabetes-related complications by improving liver histological changes (35).

### 4.3 Homocystinuria

**Figure 4:**
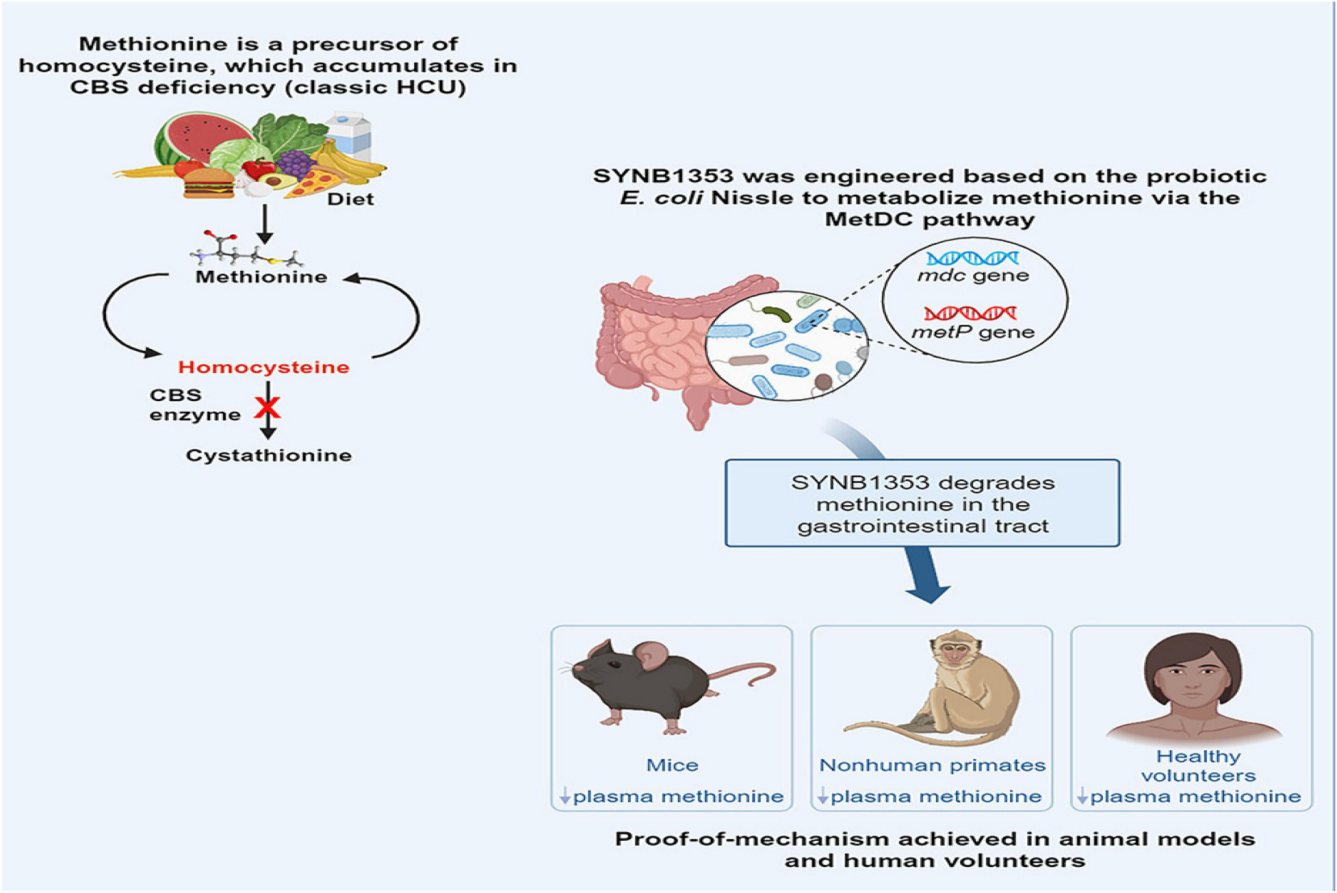
Mechanistic diagram of the effectiveness and potential therapeutic use of engineered probiotic Escherichia coli Nissle SYNB1353 in treating HCU.reproduced with permission from Ref. (46). Open Access.

Homocysteine, a sulfur-containing amino acid, is generated from the demethylation of methionine. Homocystinuria (HCU), a rare genetic disorder, stems from mutations in the cystathionine beta-synthase (CBS) gene, leading to elevated homocysteine levels due to CBS deficiency (45). HCU manifests in ocular, skeletal, cardiovascular, and central nervous system abnormalities, such as severe myopia, ectopia lentis, osteoporosis, kyphoscoliosis, and varying degrees of cognitive impairment (46). Existing treatments, which rely on diet and supplements, offer limited efficacy due to poor compliance and metabolic control (47). SYNB1353, a potential probiotic strain of EcN, is engineered to metabolize methionine in the gut, thereby reducing its conversion to homocysteine and potentially offering a promising therapeutic approach. This strain was developed through modification of Streptomyces sp., resulting in a strain that exhibits a 4.5-fold increase in 3-methylthiopropionate (3-MTP) production compared to the wild-type enzyme. Perreault et al. administered oral methionine and SYNB1353 to fasted mice and non-human primates, observing a notable decrease in serum homocysteine and methionine levels after 6 h of treatment, along with a notable increase in urinary 3-MTP and 3-MTP glycine. As shown in Figure 4. SYNB1353 has demonstrated efficacy in degrading dietary and intestinal circulating methionine in preclinical models, thus preventing its absorption and subsequent formation of homocysteine. This suggests that SYNB1353 may lead to a reduction in plasma homocysteine levels in patients with homocystinuria (48). This study explores the potential of an engineered probiotic bacterium as a therapeutic option for patients with HCU across diverse mammalian models, demonstrating promising prospects. Future discussions should delve into the adaptability of this engineered strain and achieve a balance between efficacy and safety (49).

## 5 Role of intestinal flora and its metabolites in cardiovascular and cerebrovascular diseases and regulatory strategies for engineered bacteria

With the introduction of new concepts such as the “heart-gut axis” and the “brain-gut axis”, changes in the gut microbiota and cardiovascular diseases, two seemingly unrelated conditions, have been tightly linked. Taking heart failure and CAD as examples, similar alterations in gut flora have been found in patients with both conditions, such as the microbial abundance is decreased, particularly butyrate-producing bacteria, and an increase in the circulating levels of the metabolite trimethylamine-N-oxide (TMAO). In previous studies, TMAO, often used as a key metabolite in the study of microbial metabolome alterations in cardiovascular disease, has been shown to directly stimulate platelets by enhancing intracellular calcium release, among other pathways, causing platelet hyperreactivity, thereby increasing the risk of thrombus formation (50). In a new study, it was shown that some SCFAs and branched-chain amino acid (BCCA) metabolites derived from the reactive changes of phenylalanine and tyrosine can play a role in influencing the host. An increase in the abundance of some opportunistic pathogens, such as Escherichia coli, Clostridium ramosum, Bacteroides caccae, and Eggerthella lenta, as well as a decrease in the abundance of some SCFA producing bacteria (e.g., Roseburia, Faecalibacterium and Eubacterium rectale), both of which are strongly associated with an increased risk of cardiovascular diseases (51). Therefore, increasing the biosynthesis pathway and quantity of SCFAs by gut microbiota, and relatively reducing the synthesis pathway and quantity of BCCA, may contribute to the prevention and treatment of ischemic heart disease (28).

In the area of cerebrovascular diseases, existing research mainly focuses on PD, AD, and depression. Timothy R. Sampson et al. suggest that the interaction between PD and the gut microbiota may involve the production of SCFAs by the gut microbiota to promote the maturation of microglia and the production of pro-inflammatory cytokines, thereby leading to protein production α- Synuclein (α-Syn) aggregates and activates microglia, promoting feed-forward cascade reactions, and contributing to PD progression (52). Meanwhile, the experiments of Velma T E Aho and J.P. Haran respectively demonstrated the positive impact of SCFA levels on PD (53) and the therapeutic effect of butyrate on AD through the influence on p-glycoprotein pathway (54). Another theory suggests that dysregulation of gut microbiota can activate Toll-like receptor 4 (TLR4)/tumor necrosis factor-α (TNF-α) signal pathways, which can cause neuroinflammation (55). Similarly, in AD, Kim MS et al. believe that the mechanism of action of gut microbiota may be to affect the activity of intestinal macrophages and the expression of genes related to circulating inflammatory monocytes, which can directly produce signaling molecules that act on the brain and lead to AD (56). It has been confirmed that the abundance of Bacillus subtilis and Bacteroides fragilis decreases in PD patients (57), while the abundance of Ruminococcaceae, Spirobacteriaceae, and Clostridaceae decreases in AD patients (58). The abundance of bifidobacteria and lactobacilli in AD and PD increased compared to the general population (58, 59). Several experiments have demonstrated that a variety of Bifidobacterium and Lactobacillus species can ameliorate the pathology of AD (60, 61), and the positive effects of Bifidobacterium and Lactobacillus on PD have been similarly demonstrated from *in vitro* experiments in L. Magistrelli's experiments (62). And rumen cocci can reduce the risk of developing AD and PD by maintaining the integrity of the intestinal barrier (63).

### 5.1 Cardiovascular disease

At this stage, the therapeutic applications of engineered bacteria in cardiovascular diseases are fewer compared to those for metabolic diseases and tumors, with atherosclerosis and hypertension being particularly noteworthy. Preclinical studies support a key link that the therapeutic effect of engineered bacteria on metabolic and cardiovascular diseases depends on dosage and treatment length. It also depends on the bacterial strain, therapeutic target, and disease type. In preclinical models, EcN-derived strains often need 10⁹–10¹⁰CFU per day. Preventive or therapeutic effects usually require 4–14 days of treatment (35, 64). Clinical trials were shown in Table 2.

**Table 2:** Clinical trials using modified bacteria for the treatment of metabolic/cardiovascular diseases.

Year of publicatin	Study type	Method		Results	Ref
		Control group	Treatment group		
2025	Randomized controlled trial	61 placebo for hypertension	63 FMT for hypertension	The FMT group showed a short-term reduction in office systolic blood pressure at 1 week	(65)
2024	Randomized controlled trial	15 placebo for atherosclerotic cardiovascular disease	14 Lactobacillus plantarum GLP3 for atherosclerotic cardiovascular disease	probiotic supplementation with *Lactobacillus plantarum* GLP3 significantly lowered plasma trimethylamine-N-oxide (TMAO) levels in high-risk patients with established atherosclerotic cardiovascular disease	(66)
2025	Interventional Cohort Study	20 UA + 30 HC	10 UA for Bifidobacterium	Supplementation with bifidobacterium significantly reduced serum TMAO levels in patients with unstable angina. It also led to notable decreases in total cholesterol and low-density lipoprotein.	(67)
2025	Randomized controlled trial	29 placebo for overweight/obese type 2 diabetes	29 AKK-WST01 for overweight/obese type 2 diabetes	For patients with low baseline Akkermansia muciniphila levels, AKK-WST01 successfully colonizes the gut and improves body weight, glucose, and lipid metabolism.	(68)
2025	Cohort Study Randomized Crossover Trial	All 10 CKD received both interventions (the probiotic DM02 and the placebo) in sequence	All 10 CKD received both interventions (the probiotic DM02 and the placebo) in sequence	The probiotic DM02, when administered orally, effectively reduces intestinal *E. coli* colonization and lowers plasma concentrations of its derived uremic toxin, indoxyl sulfate, in CKD patients.	(69)
2023	Randomized controlled trial	64 adults with MetS who received daily placebo capsules	66 adults with MetS who received *Lactobacillus paracasei* 8700:2	*Lactobacillus paracasei* 8700:2 significantly improved endothelial function in adults with MetS, primarily by reducing levels of atherogenic remnant cholesterol.	(70)
2022	Randomized controlled trial	8 placebo for hypercholesterolemia	8 *L. plantarum* ECGC 13110402 for hypercholesterolemia	Lactobacillus plantarum ECGC 13110402 significantly improved key cholesterol markers (TC, LDL-C, apoB, non-HDL-C) in hypercholesterolemic adults.	(71)

UA, unstable angina; HC, healthy control; CKD, chronic kidney disease; MetS, Metabolic Syndrome.

Linda S. May-Zhang et al. targeted NAPEs, precursors of N-acyl ethanolamides (NAEs), an endogenous lipid satiety factor, and engineered a commensal Escherichia coli strain (Nissle 1917) to achieve its production *in vivo*, thereby preventing or ameliorating atherosclerosis. The NAPE synthesized by this bacterium can compensate for the reduced intestinal biosynthesis of NAPEs due to the chronic patient's high-fat diet, and this compensatory effect was demonstrated in mouse models to partially restore NAE levels in mouse tissues and significantly reduce key markers of cardiometabolic diseases, such as hepatic steatosis, inflammation, and necrotic atherosclerotic plaque formation in these mice (22). For hypertension, Xin-liang Wang's team transformed Clostridium butyricum with pMTL1, a recombinant plasmid encoding the hGLP gene, to construct engineered Clostridium butyricum pMTL007-GLP-1 (CB-GLP-1). This bacterium secreted GLP-1, allowing it to bind to GLP-1 receptors (GLP-1R) in the kidneys, thereby modulating the RAAS system to lower systolic blood pressure. In addition, it reduced the expression levels of ANP, BNP, and β-MHC in cardiac tissues and activated the AMPK/mTOR/p70S6 K/4EBP1 signaling pathway, ameliorating the detrimental effects of myocardial hypertrophy and other remodeling caused by hypertension (23). Cardiovascular drugs and engineered bacteria may exhibit bidirectional regulatory effects. This process is particularly crucial for heart disease patients on long-term medication. Drugs including lipid-lowering agents, antiplatelet agents, and nonsteroidal anti-inflammatory drugs (NSAIDs) alter intestinal pH, disrupt endogenous microbial balance, and compromise intestinal barrier integrity. Consequently, These drugs may impact the colonization, survival, and metabolic functions of engineered bacteria within the gut. Conversely, engineered bacteria can enhance drug bioavailability by optimizing intestinal barrier function. They mitigate drug-induced gastrointestinal adverse reactions by promoting the production of protective microbial metabolites and suppressing the overgrowth of pathogenic bacteria (1).

### 5.2 Neurological diseases

**Figure 5:**
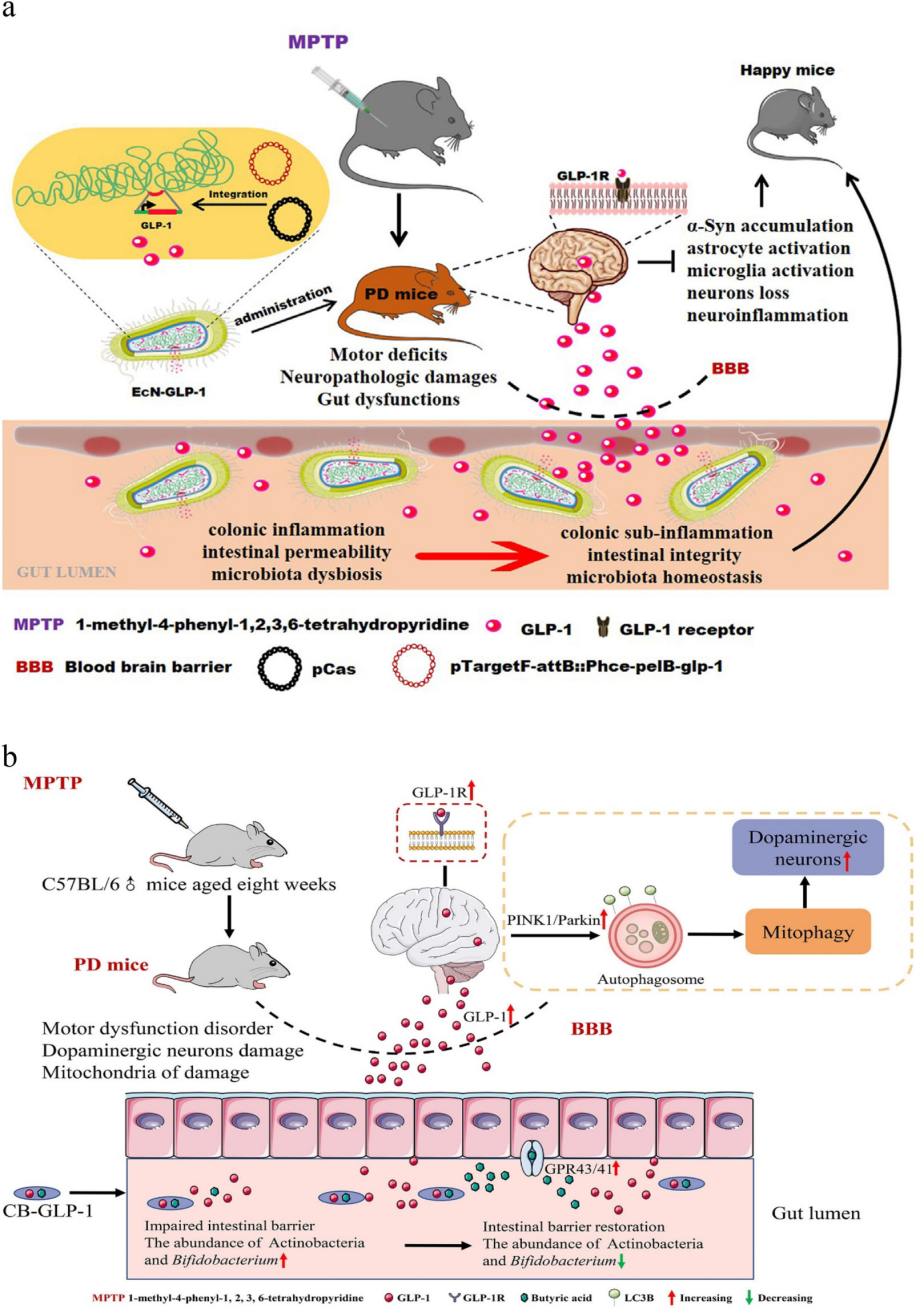
**(a)** schematic illustration of the underlying mechanisms by which EcN-GLP-1 improves Parkinson's disease. Reproduced with permission from Ref. (18). Open Access. **(b)** Schematic diagram of an engineered strain of Clostridium butyricum-pMTL007-GLP-1 in the treatment of Parkinson's disease. Reproduced with permission from Ref. (20). Open Access.

Neurological diseases, especially degenerative neurological diseases, such as PD and AD, have been the subject of a number of therapeutic programs with engineered bacteria, most of which continued to be based on the principles of bioediting different strains of bacteria to secrete GLP-1 and adjusting the intestinal flora to improve it. Heng Wu and Jing Wei et al. used CRISPR/Cas9 double plasmid system to integrate the DNA fragment containing GLP-1 cluster gene with the hce promoter and pelB signal peptide sequences into the attB site of EcN chromosome to obtain the genetically engineered strain ECN-GLP-1. This type of bacteria has been found to improve motor deficits in PD. This improvement is attributed to several mechanisms: an increase in tyrosine hydroxylase-positive neurons; inhibition of microglia and astrocyte activation; reduction of inflammation in the brain and colon; and restoration of colonic barrier function. It also reduced the relative abundance of Akkermansia and Oscillospira and increased the level of intestinal Prevotella to restore the disturbed gut microbiota (Figure 5A) (19).

Similarly, the MG1363pMG36e-GLP-1 strain from Xin Fang et al. also expresses GLP-1. In their experiments on Parkinson's mice constructed with 1-methyl-4-phenyl- 1,2,3,6 -tetrahydropyridine (MPTP), activation of astrocytes and microglia was observed in mice treated with the bacterium. The secretion of pro-inflammatory factors IL1b, IL-6 and TNF-a in PTL, TL, PTH and TH groups were significantly inhibited. And the abundance of bacteria such as Lactobacillus and Akkermansia also recovered (20). In contrast, inspired by the relationship between PD and lysosomes proposed by Andrés D Klein et al. (72), CB-GLP-1 was designed based on intracerebral mitochondrial changes in patients with PD with the aim of ameliorating motor dysfunction. It was confirmed that mice treated with this bacterium not only had significantly lower nigral α-syn levels, but also significantly increased TH and DAT levels. The expressions of Beclin-1, PINK1, Parkin, Atg7, LC3B II and LAMP-1 were greatly decreased, while the expression of p62 was elevated, thereby facilitating the removal of abnormal mitochondria by the PINK1/Parkin mitochondrial autophagy pathway. Finally, it also resumed the abundance of bifidobacteria, an important probiotic genus (Figure 5B) (21).

The production of GABA in mice can be precisely controlled by regulating red light *in vitro*. This control activates G-CSF in the gut, making it biologically active when it reaches the brain. Compared with previous studies, the major difference is the introduction of optogenetic elements. This combination of nanotechnology and genetic engineering, the construction of upconversion optogenetic micro nano system can facilitate the delivery and expression of engineered probiotics in the gut *in vitro*, and can effectively improve the precision and effectiveness of engineered bacteria (73). In addition to the above bacteria for PD treatment, there are a variety of engineered bacteria used to treat AD. One of them, MG1363-pMG36e-GLP-1, designed by Tingtao Chen, is similar to the strains described above for PD treatment and secretes GLP-1 to reduce β-amyloid and the downregulation of substances such as COX-2, TLR-4, TNF-a, and IL-1β, thereby improving diseased brain tissue and preventing lesion formation. Of course, it also significantly increased the abundance of some Lactobacillus species and reduced the abundance of pathogenic genera such as Enterobacteriaceae, Clostridium, and Clostridium Fahrenheit (74).

## 6 Limitations

At present, engineered bacteria have not been widely implemented in clinical settings, as certain inherent limitations remain inadequately addressed. Significant improvements are still required in engineered bacteria before these deficiencies can be effectively resolved.

### 6.1 Immunogenicity and regulatory discussion

Bacteria and their metabolites often trigger various immune responses in the human body, resulting in varying degrees of damage. When engineered bacteria enter the human body, the immune system identifies them as foreign invaders and initiates a multi-layered defense response. The innate immune system recognizes bacterial surface antigens, such as protein markers, thereby triggering inflammatory responses and phagocytic clearance. This can lead to side effects such as local inflammation and fever, and significantly reduces the retention time of the engineered bacteria. Simultaneously, adaptive immunity generates specific antibodies and effector T cells targeting both the engineered bacteria and the foreign proteins they express. This not only diminishes therapeutic efficacy but also may render repeated administration ineffective (75). Furthermore, interactions between certain bacterial species have been demonstrated to exacerbate infections. For example, in mono-colonized mice, Bacteroides thetaiotaomicron can acquire sialic acid from mucosal polysaccharides and produce succinate, which subsequently serves as a carbon source for Clostridium difficile during infection (80).

Gene editing may introduce genomic instability and unpredictable outcomes. A primary biosafety concern is the potential horizontal gene transfer of antibiotic resistance markers from engineered bacteria to commensal or pathogenic microbiota, thereby exacerbating antimicrobial resistance. More critically, should virulence determinants (e.g., toxin genes or adhesion factors) encoded by engineered constructs be acquired by opportunistic pathogens, they could potentiate the virulence or expand the host tropism of these organisms. Beyond the host, engineered bacteria may act as exogenous gene donors, perturbing indigenous genetic exchange networks and altering microbial co-evolutionary dynamics. Following environmental release, foreign genes that undergo HGT may persist indefinitely in the environmental gene pool, rendering their eradication or containment extraordinarily challenging. In natural environments, the frequency of HGT events is low and occurs randomly, making it challenging for conventional monitoring methods to promptly trace the pathways of gene transfer.The development of live biotherapeutics that are derived from engineered bacteria presents significant immunologic, ecologic, and regulatory considerations.

#### 6.1.1 Immunogenicity and host immune activation

The engineered bacteria have been shown to have direct impacts on the host's innate immune system via TLRs (like TLRs) through their recognition of various microbial-associated molecular patterns (MAVP's) like lipopolysaccharides and flagellin (55) (75). Activation of these pathways leads to the production of cytokines and the activation of various inflammation signaling pathways within the host (55).Although some moderate activation of the immune system will lead to increased therapeutic efficacy when used to modulate an individual's metabolic disease (14), excess activation or stimulation could lead to strong inflammatory response or worsening of autoimmune disease (39) (75).In addition, immune system responses could also be stimulated due to exposure to heterologous proteins produced by genetically engineered probiotics, such as GLP-1 analog, IL-10 (14) (19). Prolonged exposure to these proteins may result in neutralizing antibodies and decrease therapeutic efficacy and may cause later hypersensitivity.Limited long-term human immunogenicity data are available regarding genetically modified probiotics (11) (48).

#### 6.1.2 Colonization stability and ecological impact

There is a large variability in colonisation of Escherichia coli Nissle 1917 across different individual patients (76). That is, once the treatment has stopped, patients vary widely in how quickly they weaken their colonisation and how long they may remain colonised (76).A heterogeneous distribution of gut bacteria throughout the gastrointestinal tract creates a significant risk that modified bacteria will not be able to effectively colonise their preferred area (77, 78). Even if they can be successfully introduced, interactions with other bacterial species already present are subject to considerable uncertainty (28, 80).These types of ecological disruptions are likely to affect the production of short-chain fatty acids, how bile acids get processed and how signals for inflammation get generated (28, 37).

#### 6.1.3 Regulatory landscape

In the U.S., engineered bacteria designed for therapeutic purposes are normally classified as Live Biotherapeutic Products (LBPs). In Europe, they are classified as either genetically modified medicines or Advanced Therapy Medicines (ATMP) (11).Some of the required regulatory requirements for engineered bacteria include:

- Demonstration of Genetic Stability
- In the absence of Virulence Factors
- Good Manufacturing Practices (GMP) Production
- Environmental Risk Assessments

These regulatory frameworks were historically developed for biologics and cell-based therapies; therefore, they may not address the full scope of the regulatory challenges associated with engineered bacterial products, particularly in regards to engineered microbial products that can replicate in the environment following dissemination (11). Thus, it may be necessary to have long-term microbiome surveillance and post-marketing safety registries in place to support ongoing regulatory oversight of engineered microbial therapeutics in the future (11, 48).

### 6.2 Stability and limitations of clinical translation

The most commonly modified bacterium—*Escherichia coli* Nissle 1917—exhibits significant variability in colonization capacity across individuals during therapeutic administration. Within two weeks after treatment discontinuation, fewer than 50% of volunteers underwent decolonization. Six months later, only 17.5% of volunteers tested positive for this probiotic in their feces via PCR analysis (76). Moreover, the site of disease occurrence within the body also determines the choice of the most suitable therapeutic bacteria. Escherichia coli and Lactobacillus species are typically enriched in the small intestine, while Bacteroides species inhabit the cecum and colon (77). Some bacterial strains tend to colonize the mucosal layer, whereas others prefer the intestinal lumen (78). In practical applications, it is essential to select specific engineered bacterial strains based on the targeted disease to ensure high colonization stability and long-term persistence. Ensuring that engineered bacteria do not produce toxic substances or generate toxins that elicit minimal reactions and are easily neutralized remains a significant challenge. Evaluation of preclinical evidence indicates that certain bacteria may interact in complex ways with other drugs used to treat the disease. For instance, bacterial β-glucuronidase can reactivates drugs in the intestines, compromising the efficacy of the colon cancer chemotherapy agent CPT-11 and increasing its toxicity (79). Individual variations in human gut microbiota composition lead to heterogeneous responses to drug and engineered bacterial interventions. This underscores the necessity of incorporating microbiome profiling into personalized treatment strategies for cardiovascular diseases.

### 6.3 Critical appraisal of preclinical evidence and translational barriers

The preclinical evidence for the therapeutic efficacy of engineered bacteria in the treatment of metabolic and cardiovascular diseases appears promising; however, the limitations of the studies conducted to date, including a reliance on animal models, a lack of standardization across studies with regard to reporting methods, and the use of non-powered proof-of-concept studies, all impair the overall strength of the evidence currently available for the use of engineered bacteria in clinical settings (11, 28, 64).Animal Disease Models are Commonly Used. The majority of studies investigating the effects of engineered bacteria use rodent models of disease, including obesity induced by high fat diets (HFD), diabetes induced by streptozotocin (STZ), and MPTP-induced Parkinson's disease (12, 19, 35). While these models do provide some degree of insight into the pathophysiologic basis for cardiometabolic and neurodegenerative diseases, they do not completely replicate the complexity, in terms of immune diversity and microbiome diversity, of those diseases in humans (25, 28).

The characterization of dose-response relationships for engineered bacteria also requires ongoing efforts. The majority of studies to date have investigated the pharmacological effects of engineered bacteria on the central nervous system (CNS), under dosages (10⁸–10¹⁰ CFU/day) that did not employ rigorous pharmacokinetic evaluations to quantify metabolites (17, 35, 64). Currently, there are a lack of harmonized guidelines for standardizing the reporting of bacterial persistence, metabolites and exposure following administration of engineered bacteria to humans (11).The use of non-powered proof-of-concept study designs (the majority of published studies) restricts conclusions about potential for human translation, due to a lack of independent replication, blinded assessment, and sex-stratification (11, 28). Because there are well-established sex differences in the biology of metabolic and cardiovascular disease, this is a significant limitation with regards to translating these results to the clinical arena (32).

Finally, while the number of clinical investigations involving probiotics for the treatment of cardiometabolic diseases is growing (66–71), there remains a relative dearth of clinical investigations utilizing genetically engineered live biotherapeutic products (LBPs) as pharmacological agents (48, 49). In order to overcome the current translational gap from preclinical to clinical use, we must establish harmonized regulatory environments, KLAS-validated microbiome biomarkers, and scalable good manufacturing practice (GMP)-compliant production systems for LBPs (11).

## 7 Perspectives

**Figure 6:**
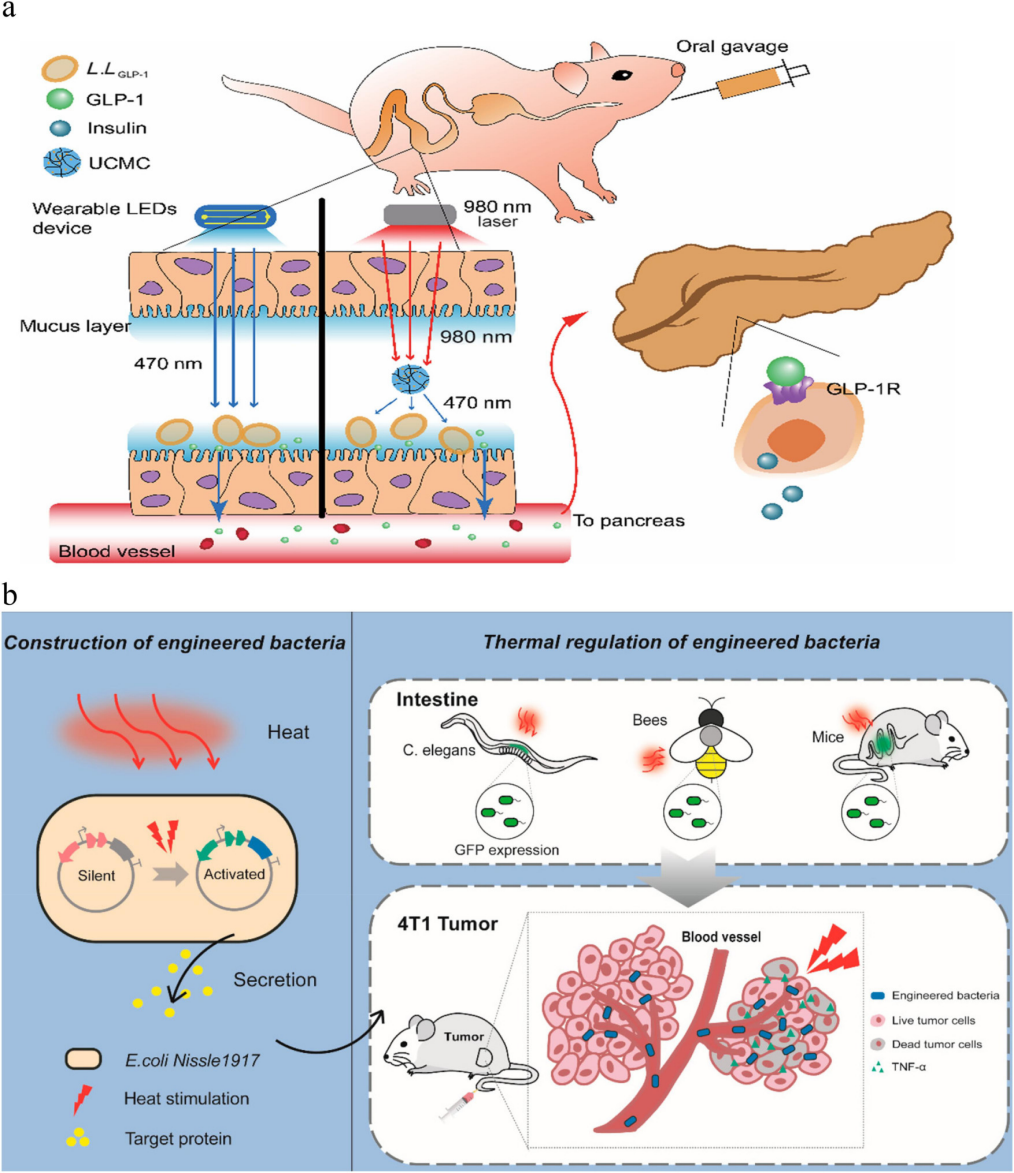
**(a)** Schematic representation of how the optogenetic probiotic system controls glucose metabolism. Reproduced with permission from Ref. (33). Copyright 2022, Springer. **(b)** Scheme of the Construction and Heat Regulation of Heat-Sensitive Engineered Bacteria. Reproduced with permission from Ref. (70). Open Access.

Engineered bacteria can be regulated *in vitro* by means of NIR light and ultrasound interventions to produce the required metabolites for customized and personalized treatment. Changing bacterial physiology and metabolism through ultrasound, light, and magnetic fields can provide temporal resolution in the minute range or higher. In recent years, engineered bacteria have made remarkable achievements in the treatment of tumors, but they are still in the research stage in metabolic diseases and cardiovascular and cerebrovascular diseases.Hanjie Wang's team combined optogenetics with engineered bacteria to design a micro- and nano-scale optogenetic system that matches the probiotic Lactococcus lactis NZ9000 (81). In experiments, rats were equipped with optical devices that provided blue light and was converted into near-infrared light by upconversion microcapsules, which enabled the secretion of intestinal endocrine hormones under external control for therapeutic purposes (81). In cancer, *in vitro* regulation of engineered bacteria can be achieved by designing a variety of schemes such as optogenetic systems [including red light regulation within the NIR (82) and blue light regulation within the NIR after conversion of the conversion material] (73, 83), wireless magnetic regulation combined with optogenetics control (84) and sonodynamic genetic systems (85), and even thermosensitive regulation that responds to thermal stimuli (Figure 6A) (86). It is worth noting that the approach of combining optogenetics with engineered bacteria, as previously mentioned by Wang's team, represents a novel idea. If the complex interaction mechanisms of optical signals in the “material device-engineered bacteria” system, specifically between the machine and biological components, can be fully elucidated, it may be possible to develop stable and convenient “optogenetically engineered probiotics.” (Figure 6B) (81) However, the regulatory system related to metabolic diseases and cardiovascular and cerebrovascular diseases diseases has not yet been fully established.

## Conclusion

The potential role of gut microbiota and their metabolites in maintaining human health and preventing diseases has been widely recognized. Engineered bacteria offer advantages such as stability, controllability, targeting, and ease of operation, making them highly promising for early and accurate disease detection and treatment. In recent years, significant progress has been made in applying engineered bacteria for metabolic and cardiovascular and cerebrovascular diseases. To advance translational applications, future research should focus on: (1) Elucidating interactions between engineered bacteria and the gut microbiota to identify inflammation-related regulatory targets; (2) Optimizing probiotic engineering strategies and exploring combined applications with nanotechnology or optogenetics; (3) Conducting large-scale clinical trials to validate safety and establish standardized treatment protocols for obesity-related cardiovascular complications. Additionally, the continuous introduction of regulatory systems such as light, ultrasound, and magnetic fields has further advanced the translational development of engineered bacteria. Interdisciplinary collaboration among microbiologists, cardiologists, and genetic engineers will help overcome translational bottlenecks. Engineered bacteria represent a transformative frontier in therapeutics and diagnostics, warranting sustained and rigorous research.
